# Trends of cross-border mobility of physicians and nurses between Portugal and Spain

**DOI:** 10.1186/1478-4491-11-36

**Published:** 2013-07-31

**Authors:** Claudia Leone, Cláudia Conceição, Gilles Dussault

**Affiliations:** 1 Institute of Hygiene and Tropical Medicine, Nova University, Lisbon, Portugal; 2 Life and Health Sciences Research Institute (ICVS), School of Health Sciences, University of Minho, Campus de Gualtar, 4710-057 Braga, Portugal; 3 ICVS/3B’s - PT Government Associate Laboratory, Braga/Guimarães, Portugal

**Keywords:** Cross-border mobility, Health workers, Portugal, Spain

## Abstract

**Context:**

Health workforce cross-border mobility has an impact not only on individual health workers, but also on how health services are organized, planned, and delivered. This paper presents the results of a study of current mobility trends of health professionals along the borders between Portugal and Spain. The objective was to describe the profile of mobile physicians and nurses; to elicit the opinions of employers on mobility factors; to describe incentive policies to retain or attract health professionals; and to collect and analyse employers’ opinions on the impact of this mobility on their health services.

**Methods:**

Phone interviews of key informants were used to collect relevant data. The interviews were conducted during December 2010 and January 2011 in health organizations along the border of the two countries. In Portugal and Spain, four and 13 organizations were selected, respectively. Interviews were obtained in all the Portuguese organizations and in four of the Spanish organizations.

**Results:**

Findings suggest that cross-border mobility between the two countries has decreased. From Spain to Portugal, mobility trends are mainly of physicians who seek professional development in the form of specialization, the availability of positions, better salaries, and the perceived good living conditions. The mobility of nurses lasted until 2008, when reforms improved working conditions in Spain and contributed to reversing the flow. Since then, there has been an increase of Portuguese nurses going to Spain seeking better working conditions or simply a job. Portuguese nurses as well as Spanish physicians are well considered in terms of professionalism and qualifications by their Spanish and Portuguese hosts, respectively.

**Conclusions:**

There is a deficit of valid data on the health workforce in general. The present study allowed further exploration of the reality of the mobility trends between Portugal and Spain. At present, the mobility trends are mainly of Spanish physicians to Portugal and Portuguese nurses to Spain. There is a consensus on both sides of the border that the benefits of migratory flows are much greater than the limited problems (for example, language and salary differences) that they may bring.

## 

This paper presents the results of a study that documented current trends of mobility of health professionals along the borders of Portugal and Spain which share a border of 1,215 km from North to South. The specific objectives are to characterize the profile of health professionals who cross the border for working purposes; to elicit the perceptions of employers on mobility factors; to describe the recruitment processes of health professionals in selected health organizations along the border; to describe incentive policies to attract or retain health professionals and to collect and analyse employers’ opinions on the impact of this phenomenon on health services systems.

## Context

Portugal’s National Health Service (NHS) offers universal coverage, is comprehensive, and is predominantly financed through general taxation. The NHS is the main provider of healthcare for which user fees are applicable to persons above a certain income level, but there is a growing private sector, as well as public-private partnerships in the hospital sector. The management of the NHS mostly takes place at central and regional levels. The Ministry of Health is responsible for the definition, implementation, and monitoring of national health policy objectives, for the strategic management of funds, and employs service providers. There are five Regional Health Administrations (RHA), which have the mission of ensuring access to quality healthcare to the population under their jurisdiction and to implement the National Health Policy. Under the RHAs, which are directly accountable to the Ministry of Health, there are local health units (*unidades locais de saúde*), groups of health centers (*ACES*; *agrupamentos de centros de saúde*), and hospital centers (*centros hospitalares*). Local Health Units are groupings of health centers and hospitals, usually composed of two or more previously autonomous organizations, functioning under a common management structure. Seventy-three groups of primary care health centers have been created in 2009 to improve efficiency through better coordination and sharing of resources. In 2012, the Ministry of Health has announced that the number of *ACES* would be reduced to 46, as part of the austerity program which the Government of Portugal is conducting. The creation of more local structures was presented as an effort towards decentralization, but in the absence of financial autonomy, the dependency on RHAs remains strong. Hospital centers are groupings of at least two hospitals located in the same region (although frequently involving hospitals in different towns). From those hospitals most of them acquired, in the last 10 years, a legal status of financial and administrative autonomy (*EPE*, *Entidades Públicas Empresariais -* Public Autonomous Organizations). Only Local Health Units and EPE hospitals or Hospital Centers have the autonomy to directly hire health personnel [1].

The main employer of health professionals is the NHS (Tables 1 and 2) [1,2]. The private sector is growing, and is becoming an important employer, with an undetermined number of professionals working in both the public and the private sectors. There is no monitoring of dual employment nor of mobility of health professionals [1,3].

**Table 1:** **Number of physicians in Portugal**, **2003**, **2007**, **2008**, **2009, and 2010**

**Physicians ( *n *)**	**2003**	**2007**	**2008**	**2009**	**2010**
Total registered	34.981	38.399	39.470	40.664 [4]	42.031[4]
Foreign physicians registered	2.987	3.656	4.397	3.842 [4]	3.937 [4]
Spanish physicians registered	1.757 [5]	1.971 [5]	NA	NA	NA
Total physicians in NHS	24.444 [2]	24.831 [2]	25.106 [2]	23.007 [6]	23.324 [6]
Spanish physicians in NHS	1.090 [2]	913 [2]	799 [2]	681 [7]	696 [7]

Detailed description: this table shows the number of physicians registered in Portugal, total, foreign and Spanish, total and Spanish physicians working in the Portuguese NHS, 2003, 2007, 2008, 2009, and 2010.

NA, not available; NHS, National Health Service.

Source: Medical Council and references number [2,4-7].

**Table 2:** **Number of nurses in Portugal**, **2003**, **2007**, **2008**, **2010, and 2011**

**Nurses ( *n *)**	**2003**	**2007**	**2008**	**2010**	**2011**
Total registered	43.978	50.955	56.859	62.566	64.535
Foreign nurses registered	2.298	2.135	2.037	2.005	1958
Spanish nurses registered	1.815	1.362	1.232	1.154	1133
Total nurses in NHS	35.310 [2]	38.260 [2]	38.561 [2]	39.522 [6]	39.686 [6]
Spanish nurses in NHS	1.427 [2]	413 [2]	319 [2]	310 [7]	NA

Detailed description: this table shows the number of nurses registered in Portugal, total, foreign and Spanish, total and Spanish nurses working in the Portuguese NHS, 2003, 2007, 2008 2010, and 2011.

NA, not available; NHS, National Health Service.

Sources: Nursing Council and references number [2,6,7].

In Spain there is also a NHS providing universal coverage, almost fully funded from taxes and predominantly within the public sector. Access to services is free of charge at the point of delivery [8]. Planning and the delivery of health services are done at the level of the Autonomous Communities. There are 17 regional ministries or departments of health, with jurisdiction over the organization and delivery of health services within their territory. Health expenditure is mainly determined by these regional administrations, which are accountable only to their regional parliament and are not hierarchically linked to the national level [8].

The national Ministry of Health and Social Policy has only the authority over legislation on pharmaceuticals and is the guarantor of the equitable functioning of health services across the country. The Ministry became in 2009 the national authority in the field of social and dependency care [9]. There are also local authorities in some provinces and municipalities that have the competency over sanitation and which collaborate in health services provision and direct management of ‘residual’ public health and community services [8].

### The medical and nursing workforce

In Spain [10] and in Portugal [11-14] there is a deficit of updated and valid data on the health workforce in general. Multiple sources offer partial views of reality, as illustrated by the list of sources of data available in Portugal (Table 3). In the next two sections, data available will be presented and discussed.

**Table 3:** Information sources: characteristics and limitations

**Health workforce**	**Variables**	**Comments**
**Information sources**		
Medical Council (*Ordem dos Médicos*)	Name	• Only demographic characteristics and geographical distribution based on the area of residence.
	Residence	
Registration	Age	
	Year of graduation	• Lack of information on register updating as a result of long-term absence, death, or retirement.
	School of graduation	
	Specialization (most of the times)	
Nursing Council (*Ordem dos Enfermeiros*)	Name	• Demographic characteristics and geographical distribution based on place of work.
	Address	
Registration	Age	• Lack of information on register updating as a result of long-term absence, death, or retirement.
	Year of graduation	
	School of graduation	
	Place of work	
	Specialization	
Dental Council (*Ordem dos Médicos dentistas*)	Name	• Demographic characteristics and geographical distribution based on the area of residence and work.
	Address	
Registration	Age	
	Year of graduation	• Lack of information on register updating as a result of long-term absence, death, or retirement.
	School of graduation	
	Place of work	
	Residence	
	Nationality	
Pharmacy Council (*Ordem dos Farmacêuticos*)	Name	• Demographic characteristics and geographical distribution based on the area of residence.
	Address	
Registration	Age	• Lack of information on register updating as a result of long-term absence, death, or retirement.
	Sex	
	Professional area	
INE, Statistics Portugal	Resident population according to sector of activity.	• The national population censuses provide the broadest picture available of the workforce in general and of the healthcare workforce
National population census		
		• Last one in 2011.
INE, Statistics Portugal		• INE uses the databases from the administrative proceedings of professional association (Councils and Labour unions).
Health workforce statistics		
Central Administration of the National Health Service, Ministry of Health (*ACSS Administração Central do Sistema de Saúde*)	Annual data	• Personnel working in the National Health Service (mainland, not including the islands of Azores and Madeira).
	Nationality	
	Profession	
	Age	• Comparison of data in temporal trends is possible only for national aggregated data.
	Sex	
Health personnel database	Place of work (Health Region, hospital, primary healthcare)	
	Professional specialization	
Ministry of Labour and Social Affairs - Strategy and planning office (*Ministério do Trabalho e da Segurança Social* - *Gabinete de estratégia e planeamento*)	Annual data	• The legal imperative of providing the information exists for all the enterprises but the practice is that the public ones do not perform this duty regularly.
	Workers nationality	
	Remuneration	
	Contract types	
Personnel list from all the enterprises with workers (*Quadro de pessoal*)	Remunerated number of hours by month, normal and supplementary hours	
Portuguese Foreigners and Border Affairs Department (*Serviço de Estrangeiros e Fronteiras*)	Nationality	• Information on foreigners; the declared profession maybe different from that the person had on its country of origin.
	Profession that allows to have means of living at the moment of request of residency	
Request of residency in Portugal	Age, sex	
	Level of education	

#### ***Portugal***

In 2010, the number of physicians per 1,000 population in Portugal was above the EU27 average (3.8 in Portugal; 3.4 EU27 average). The density of nurses was well below the EU27 average (5.7 per 1,000 population *versus* the EU27 average of 7.9); Portugal has one of the lowest ratios of nurses to physicians in the EU (1.5 *versus* 2.5 in EU27) [1].

Foreign physicians and nurses represent a small proportion of the total of professionals working in the Portuguese NHS: 6.85% of physicians and 1.19% of nurses in 2008 [2]. There has been an increase of foreign workers from 313 in 1994 to a peak of 4.490 in 2004 [14,15]. Until 1999, the main source of foreign health workers was the five African countries with Portuguese as official language (former Portuguese colonies until 1975), but after 2002 Spain became the main source country. Last available data show that 46% (*n* = 799) of foreign physicians and 49% (*n* = 319) of foreign nurses were from Spain [14,15] (Tables 1 and 2).

#### ***Spain***

In Spain, the number of all categories of health professionals per 1,000 population has increased steadily over time, principally nurses, dentists, or pharmacists, whereas the physicians/population ratio has grown more slowly. In 2010, there were 3.8 registered physicians per 1,000 as in Portugal, and 4.9 nurses, a figure well below EU27 average [16]. National figures differ slightly from those provided by the World Health Organization (WHO) or even by regional authorities due to the absence of a central registry of health professionals [8]. There is no national data registry of health professionals but rather 17 regional registries. The National Health Service Human Resources Commission has the task of planning for the needs of the NHS [17]. Various autonomous communities have started to hire doctors trained abroad, mainly from Latin America, in certain specialties for which they expect a shortfall, but no figures are available.

**Figure 1:**
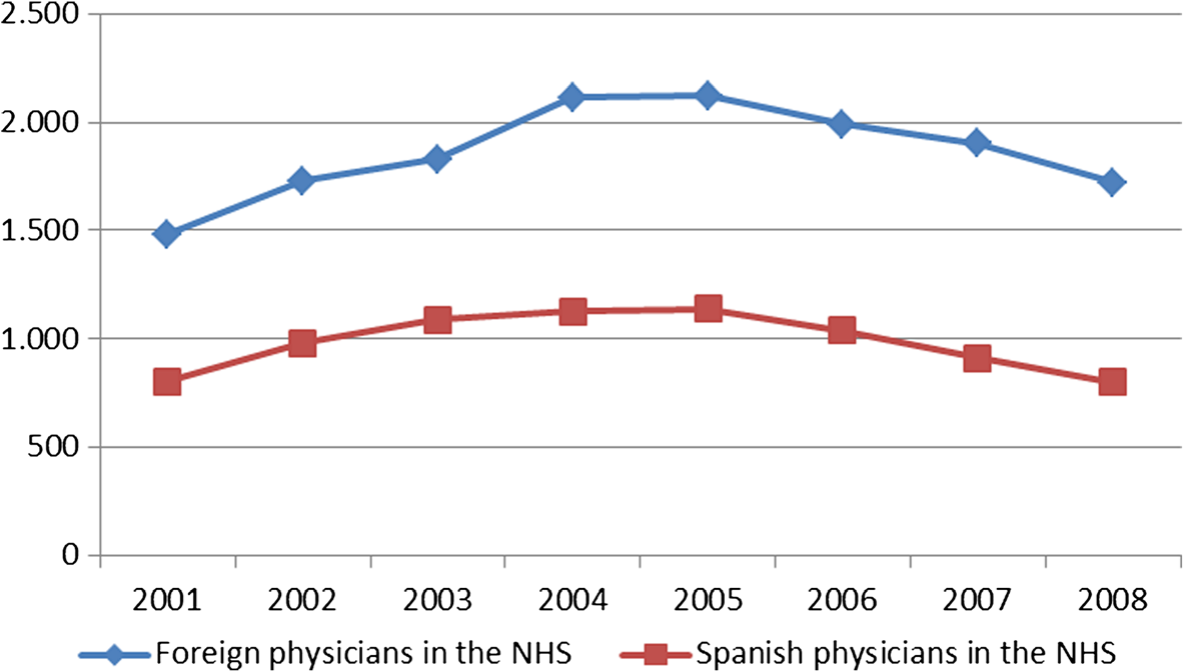
**Total number of foreign and Spanish physicians in NHS, Portugal, 2001–2008.** Source: Reference number [2].

**Figure 2:**
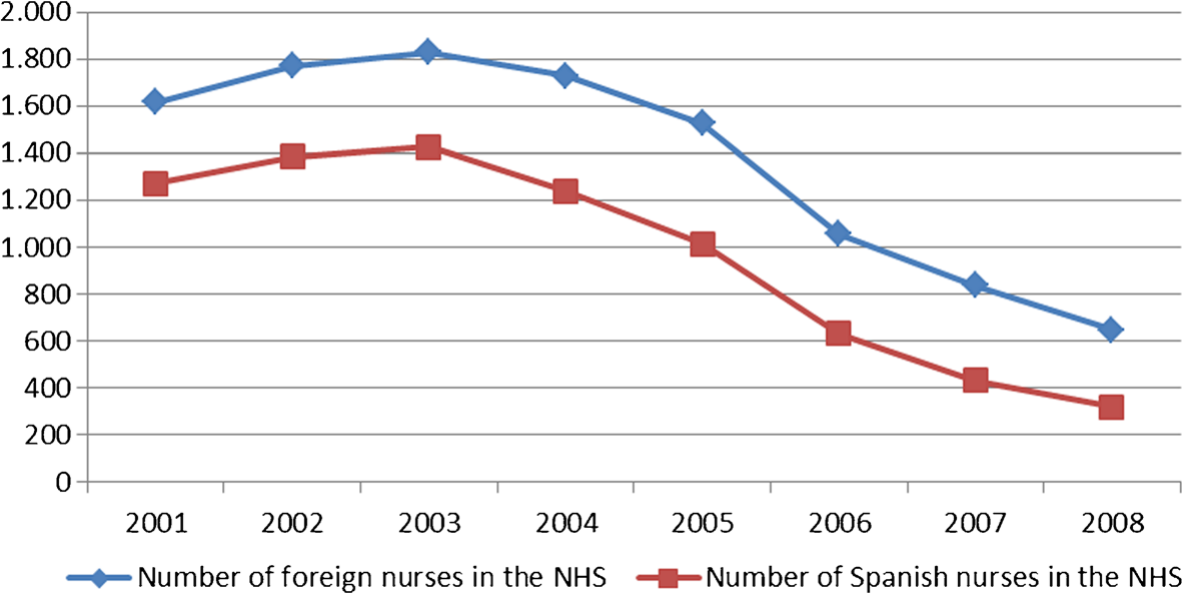
**Total number of foreign and Spanish nurses in NHS, Portugal, 2001–2008.** Source: Reference number [2].

The evolution of the total number of Spanish physicians and nurses in Portugal is related to the evolution of Spanish workers mobility (Figures 1 and 2). The highest peak for physicians was achieved in 2005 (1,140 physicians, Figure 1) and in 2003 for nurses (1,427, Figure 2 and Table 2).

## Methods

Information on the mobility of professionals was collected by telephone interviews of key informants. This method was selected to minimize time and financial costs due to distances between health units along the border. Key informants were human resources managers or their representatives.

The study units were health organizations located along the border between the two countries. The distances between the cities from both sides of the border are typically around 100 km (for example, 101 km from Badajoz to Évora, 112 km from Huelva to Faro).

The map of Hospitals EPE produced by the Portuguese Ministry of Health was used to identify NHS hospitals located in the Portuguese regions bordering Spain. In the case of Portugal we chose hospitals EPE (*Entidades Públicas Empresariais* - Public Autonomous Organizations) [18] because they have the autonomy to directly hire health personnel. All the organizations in the border region were included: two were Local Health Units and two were Hospital Centers, each composed of two or more organizations sharing the same administration and human resources department (Table 4).

**Table 4:** Organizations included in the survey

**Portuguese local health units and hospital Centers**				
**Regional health administration**	**Local health units and hospital centers**	**Health units included**	**Beds ( *n *) (2010)**	**Physicians ( *n *) (2010)**
North	Nordeste Hospital Center	Hospital of Bragança	435	143
		Hospital of Macedo de Cavaleiros		
		Hospital of Mirandela		
Center	Cova da Beira Hospital Center	Hospital of Fundão	342	153
		Hospital of Pêro da Covilhã		
Alentejo	Local Health Unit of Norte Alentejano	Hospital of Elvas	276	189
		Hospital of Portalegre		
		Group of health centres (ACES) of São Mamede		
		Group of health centres (ACES) of Caia		
Alentejo	Local Health Unit of Baixo Alentejo	Hospital of Beja	230	204
		Hospital of Serpa		
		Group of health centres (ACES) of Baixo Alentejo		
Spanish hospitals				
Autonomous Community	Hospitals	Private / public	Beds (*n*) (2010)	
Castilla y León	Hospital of Santisima Trinidad, Salamanca	Private	118	NA
Galicia	Sanatorio of Santa María, Pontevedra	Private	71	NA
Andalucía	Juan Ramón Jimenez Hospital, Huelva	Public	631	NA
Galicia	Miguel Dominguez Hospital, Pontevedra	Private	138	NA

Note: Total Portuguese NHS capacity: 20,273 beds [19]; Total beds available in Spain, public and private sector: 160,981 [8].

NA, not available.

Source: [19,20].

The selection in Spain was based on the Hospital National Catalogue, which publishes a list of public and private (for-profit and not-for-profit) hospitals [21]. The 26 health units, out of a total of 87, located in the Autonomous Communities bordering Portugal (Galicia, Castilla y León, Estremadura, and Andalucía) were selected. Telephone contacts were made to identify hospitals with >20 beds and which employed Portuguese health professionals: the final list included 13 units. The excluded units include a home for the elderly, two which did not meet the number of beds criterion, two which shared the same administration and human resources department of selected hospitals, and eight with no Portuguese health professionals on their staff.

Further telephone contacts were made to identify the Human Resources manager and to request an interview. Emails were sent with information on the project and with the interview schedule. The correspondence with Spain was in Spanish.

An interview schedule was designed to cover the following topics: (1) characterization of the population of health professionals from the other country working in the organization (categories of professionals, specialties, whether they live in the country of work or not, evolution of number of contracted over time); (2) reasons for mobility; (3) reasons for hiring foreign professionals; (4) consequences of mobility (for the organization, for the country, for the specific region); and (5) incentives: local and organization level incentive policies to stimulate recruitment. The interviews were conducted during December 2010 and January 2011. They were recorded and summarized according to thematic areas for analysis purposes.

## Results

All pre-selected organizations in Portugal responded (Table 4) and four in Spain (Hospital of Santisima Trinidad, Salamanca; Sanatorio of Santa María, Pontevedra; Juan Ramón Jimenez Hospital, Huelva; Miguel Dominguez Hospital, Pontevedra). No response was obtained from the other nine Spanish hospitals, even after repeated contacts by telephone. These hospitals are general district hospitals, which do not offer care in some specialty areas such as cardiothoracic surgery or neurosurgery. In Portugal, this type of specialized care is provided in central hospitals located in the metropolitan areas of Lisbon, Oporto, and Coimbra.

Mobility was mainly of Spanish physicians coming to Portugal and of Portuguese nurses going to Spain. Tables summarizing the findings are provided as Additional file 1.

### Spanish physicians in Portugal

#### ***Profile***

Interviews in Portugal indicate that Spanish physicians who cross the border are a mix of general practitioners and specialists. Many work in emergency departments, as these are more likely to have unfilled positions. The Nordeste Hospital Centre reported 18 Spanish physicians (13% of total), 12 in the region of Bragança and 6 in Mirandela; these included 13 specialists, three general practitioners, and two residents. The Cova da Beira Hospital Centre employed seven nurses and 12 physicians from Spain (eight specialists and four residents, 8% of total). The Norte Alentejano Local Health Unit reported 44 physicians (23% of total); 19 (17 specialists and two residents) were working in the Hospital and the others (23 general practitioners and two residents) in health centers. Further south, the Baixo Alentejo Local Health Unit reported 29 Spanish physicians (14% of total), including 20 internists and general practitioners and nine residents. Most, though not all lived in Portugal; those crossing the border and returning home were usually working in emergency services.

#### ***Reasons for mobility***

According to informants, there has been an immigration of Spanish physicians to Portugal for >10 years. Access to specialization, the availability of positions, better salaries, and better technical and organizational conditions (access to surgery rooms, team work, accredited institutions) were the main motivating factors for crossing the border. Informants welcomed this immigration which augmented the supply of specialized and primary care services.

A Portuguese hospital manager mentioned that in the past there was also a significant flow of nurses from Spain to Portugal. This trend lasted until 2008, when reforms which improved working conditions in Spain contributed to reversing the flow.

There are no incentives in place to attract Spanish physicians, but the possibility of topping up their salary income with extra hours in the emergency room is said to be a factor of attraction. Spanish professionals are also said to seek job opportunities in Portugal also for its perceived good living conditions.

#### ***Reasons for hiring***

Spanish professionals are well considered and received in Portugal. They have been an important pool of resources to help meet the demand for services in border regions, which are considered remote and isolated by Portuguese professionals.

Informants expressed high levels of satisfaction with the work of Spanish physicians though they noted the potential negative impact of language differences on the doctor-patient relationship, and the difficulty to harmonize titles and degrees (an issue which is disappearing with the implementation of the Bologna norms). The latter creates problems in assigning staff to a specific professional cadre, and at times creates situations in which persons with similar professional profiles and occupying the same position, receive different salaries.

### Portuguese nurses in Spain

#### ***Profile***

In Spain, at the time of interviews, two nurses from Portugal worked at the Hospital General de la Santisima Trinidad in Salamanca, 12 at the Sanatorio of Santa Maria in Pontevedra, and one at Juan Ramón Jimenez Hospital in Huelva intermittently. The Miguel Dominguez Hospital in Pontevedra started to recruit Portuguese nurses in 2007, and had hired 23 since then, who stayed for periods of between 1 and 2 years.

There were no reports of Portuguese physicians working in these organizations in Spain.

#### ***Reasons for mobility***

Portuguese nurses look for better working conditions, or simply a job, as there is unemployment and under-employment of nurses in their country. Informants indicated that Portuguese nurses were basically attracted by the availability of positions and that they were sought after for their good qualifications and positive attitudes towards work.

#### ***Reasons for hiring***

Spanish interviewees reported that since 2008 there has been an increase of Portuguese nurses going to Spain for periods of 1 or 2 years, but no specific reasons were implied for hiring Portuguese nurses besides the need to respond to the sporadic demand.

There was no report of incentive policies.

No consequences were reported. In Spanish interviewees’ opinion, Portuguese nurses are well considered and recognized as equals by their Spanish hosts in terms of professionalism and qualifications.

**Figure 3:**
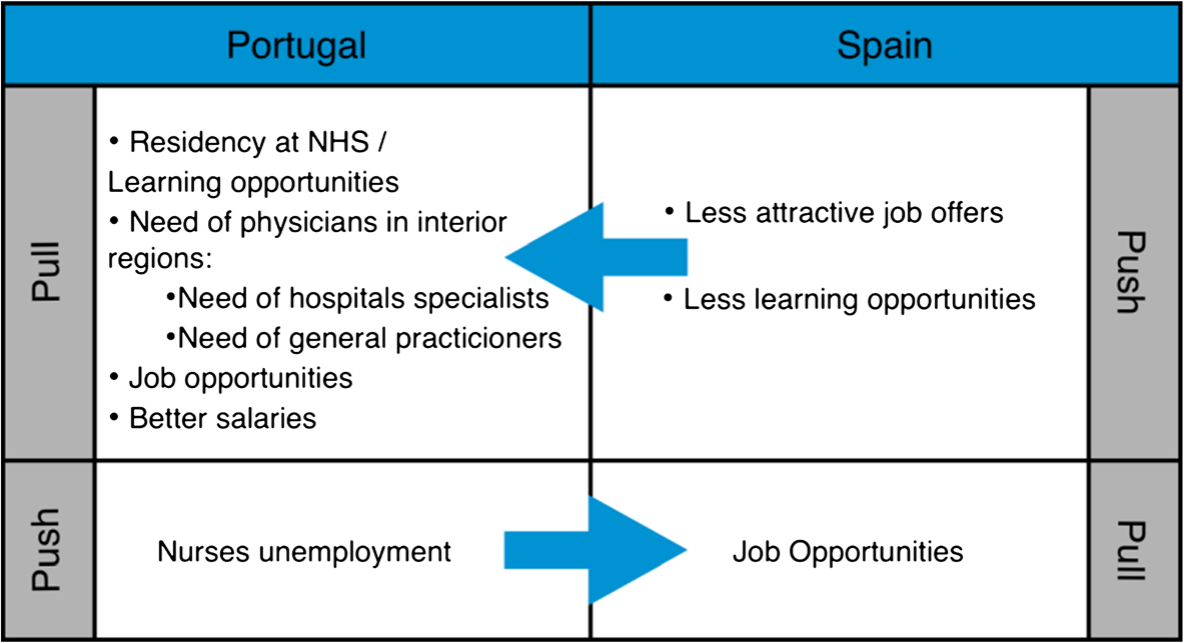
Summary of push and pull mobility factors.

In general, the main reasons that bring physicians from Spain to seek work in Portugal and nurses from Portugal to go to Spain are similar. The main pull factor for professional groups is job opportunities, and for physicians, the possibility of doing a residency and access to better learning opportunities and salaries are also factors of attraction (Figure 3).

## Discussion

This was a first attempt to characterize the mobility of health professionals across the border of the two countries. The study was conducted from Portugal and from a Portuguese perspective. Further studies with a Spanish counterpart would most certainly constitute an added value to the knowledge on cross-border mobility between the two countries.

The data obtained is not accessible through information publicly available by the Ministry of Health, Regional Health Administrations or the organizations themselves. The design of the study allowed the authors to complete it in a context of time and budget constrains (the study was performed as part of the European Union funded project ‘MoHProf - Mobility of Health Professionals’ [22]) and to obtain new data and perceptions.

The results have to be interpreted in light of limitations relative to the number of interviewees (one per organization) and to the absence of validation against the organizations’ registries. Also, the limited number of Spanish respondents (four out of 13 eligible hospitals) means that only a partial picture of the phenomenon of cross-border mobility is available.

The 103 physicians identified as working along the border in Portugal represented about 15% of Spanish physicians working in the Portuguese NHS (Table 1) and 15% of physicians in the organizations surveyed [19], which themselves accounted for 6% of the total bed capacity of the NHS (Table 4). This is not quantitatively large, but in isolated regions of a small country like Portugal, they may have a significant impact on access to services and thereby have important policy implications.

### From receiving to sending country

Interviews conducted with informants from professional councils and trade unions in a septe study and statistical data on foreign health workers in Portugal suggest that the country evolved from receiving foreign health professionals, mainly from Spain and to a lesser extent from the Portuguese-speaking African Countries (PALOPs) and Brazil, to sending professionals to other countries [3,22]. Unemployment, precarious employment, and the difficulty to access specialty training were the main reasons for Spanish physicians to come to Portugal. The number of unemployed physicians in Spain was estimated at 24,000 physicians (22% total) in 1999 [23]. Emigration was estimated at 900 physicians per year between 2002 and 2006 [10,24], with the USA [10], Portugal, and United Kingdom [24,25] as the main destinations. Access to postgraduate specialty training was limited as the number of candidates was higher than available positions in the 1990s [26,27]. Portugal became an option for those unable to access specialty training [26-28]. The opening of positions in the public sector in Spain from 2008 onwards triggered the return of physicians.

Unemployment was the main reason for Portuguese nurses to go to Spain. The Portuguese Nursing Council estimated, in November 2012, that 20% of total nurses are unemployed or underemployed [29]. The time between the end of their studies and their first job has increased in the last 3 years. In 2009, 29.7% of them waited 3 months to start their professional activity. In 2010 and 2011 the mode was 3 to 6 months (29.1% in 2010 and 28% in 2011) [30]. Between January and October 2012, the Nursing Council received 1,035 requests for certificates of qualification, a document needed to work in a foreign country [29].

Although one interviewee stated that the flow from Spanish nurses to Portugal has decreased after 2008, NHS data (Figure 2 and Table 2) show a trend to decrease starting in 2003. Also for physicians (Figure 1 and Table 1) this trend started before, in 2005. Previous work on physician’s geographical distribution (in 2008) had showed that being Spanish increased the odds of being based outside the Lisbon and Oporto metropolitan areas [31]. This might indicate that Spanish physicians working along the border are not returning to Spain at the same pace as those working in the biggest urban areas.

### Cross-border mobility impact

Cross-border mobility impacts not only on individual health workers, but also on how health services are organized, planned, and delivered, as well as on their quality and efficiency. Like most healthcare systems, those of Portugal and Spain are challenged by workforce imbalances [32]. This is the case when the labor market cannot absorb professionals willing to work or when working conditions and career development opportunities are not seen as satisfactory. This may trigger various types of mobility: within the healthcare system from one type of job or of organization to another one, movement between regions, emigration, or even exit of the health sector. Mobility along the borders of neighboring countries is an attractive option, particularly when there are cultural affinities and when it allows maintaining close links with the country of origin, like is the case when a professional works in another country but continues living in his own. For health organizations, access to a foreign workforce close by can be seen as an opportunity to expand their recruitment area. Two recent studies [33,34] have documented trends similar to those observed in Portugal and Spain; mobility along the borders of Austria and Hungary, Belgium and France, Belgium and the Netherlands, France and Switzerland, are examples. In our study, key informants stated that the benefits of employing professionals from across the border were much greater than the problems that they may entail, such as language differences or the difficulty to harmonize titles and degrees.

### The situation after 2011

The interviews were conducted at the end of 2010 and beginning of 2011. More recent developments show that Spain and Portugal’s health sector had to bear the consequences of the economic crisis. Both countries have applied austerity measures that have greatly affected the health sector.

In the case of Portugal, there has been budget and personnel reduction of public hospitals [35], doubling of user fees (applicable to households above a certain level), cuts in reimbursement of medicines, and exclusion of services from insurance coverage [36].

Efforts to achieve fiscal balance have also affected both countries workforce, including salary cuts and freezing of promotions and a reduction of personnel replacement rates [37,38]. In Portugal, public sector health professionals’ salaries have become less competitive with private sector’s ones, which has stimulated transfers to that sector [39]. In Spain, surgical and clinical activities also suffered a significant reduction causing an increase of workload for health professionals (mostly of nurses) [38].

In general, data on health workers employed in public services do not show reduction in total numbers neither in Portugal nor in Spain [40]. Nonetheless, there are reports of mobility of Spanish and Portuguese health workers to the UK, especially from nurses [41]. There is also some new evidence, although not official, of health professionals going to Latin America as an alternative to European Union countries. There are no signs that the economic crisis will end soon in neither country, and there is a need to monitor the further consequences on the mobility of its health workforce, which at present no mechanism is set to do.

### The regulation of cross-border mobility

The responsibility for planning and regulating the health workforce in Portugal is shared between the Ministries of Health and of Education and Professional Councils. There is no explicit policy or formal strategic plan for the development of the health workforce in Portugal, even though this has been identified as a need [13]. The National Health Plan 2004–2010 recommended the development of a human resources for health plan, but no steps were taken in that direction. A position paper commissioned in preption of the NHP 2011–2016 proposed a strategic framework, including objectives for the 2011–16 period to be discussed by stakeholders and validated by further studies [11]. The discussion has yet to take place.

In Spain, each regional government usually has their functions regarding health divided between a health department and the regional health service. The health department is responsible for regulation and strategic planning, while the regional health service is responsible for operational planning, management of the services network, and coordination of healthcare provision.

The health system also draws on the input of a number of other ministries; in the case of human resources regulation, the Ministry of Education, is responsible for the regulation of health professionals’ undergraduate training and, in association with the Ministry of Health and Social Policy, of postgraduate training and human resources planning.

In both countries, the main regulatory mechanisms of physician’s numbers are the *numerus clausus* and the exam to access postgraduate specialty training. In Spain, in spite of unemployment and emigration of physicians, there are claims that there is a deficit of physicians [10,24,42,43]. In Portugal, the *numerus clausus* for entry into medicine has fluctuated from 805 in 1979 to 272 in 1984, and then gradually increasing up to 1,400 in 2007 [44] and to 1,517 in 2012 [45]. The basis on which the *numerus clausus* and the number of specialty places are established are not explicit. There is an unmet demand for medical training which brings unsuccessful candidates to seek training opportunities abroad. An estimated 1,300 young Portuguese study medicine in Spain, Hungary, the Czech Republic, Slovakia, among other countries [46]. As in Spain, there is no consensus on whether there are enough, too few or too many physicians in the country. The absence of strategic thinking and planning results in a reactive policy of successive decreases and increases of *numerus clausus* and places for specialty training.

In Portugal, there is no policy to attract national physicians and nurses to regions of the interior. In fact, the Ministry of Health has opted for recruiting foreign health workers through bilateral agreements with Latin American countries, starting with Uruguay, and then with Cuba, Colombia, and Costa Rica, to fill positions in isolated or remote areas. This *ad hoc* strategy may be linked to the fact that Portugal’s health labor market is no longer attractive for its own professionals as indicates the success of recruitment agencies offering more attractive job opportunities to work in richer European countries and in places as far like the Gulf States and Australia [47].

## Conclusion

The present study explored the mobility trends along the borders between Portugal and Spain to understand better the motives for which professionals decide to work on the other side of the frontier. Trends have been of Spanish physicians to Portugal and of Portuguese nurses to Spain. The number of professionals involved is small, but the impact on health organizations along the borders is important. The motives for crossing the border are mostly related to greater availability of work in the destination country.

In the European Union, there is freedom of movement of workers, and Member States that are geographically contiguous need to take into account as they plan their workforce the possibility that health professionals may decide to cross their borders for work. Portugal only has borders with Spain, with which it has some cultural affinities, which makes migration easier. To our knowledge, no national (or regional in the case of Spain) policy exists to ‘manage’ these flows. Key informants did not even see the need for such policy and seemed satisfied with the current situation. This not to say that it is not needed when the whole health workforce and health system is considered; for instance, migrant professionals are a gain for the receiving country, but a loss of investment for the sending one. When they reach a significant level, migratory flows are a symptom of labor market deficiencies and of poor planning. The question of the need or opportunity for governments to intervene is raised and harmonization of policies between neighboring countries may be opportune. Whether this will happen depends on the political willingness to address health workforce issues, such as that of mobility. To our knowledge, neither in Portugal or Spain there is an explicit health workforce policy and any form of monitoring and analysing mobility trends, such as exits of health professionals from the health sector or from the country. In that sense, the issue of managing the consequences of cross-border mobility is as much political as it is technical.

## Competing interests

The authors declare no competing interests.

## Authors’ contributions

CL, CC, and GD designed the study and wrote the paper; CL and CC conducted interviews and analyzed data. All authors read and approved the final manuscript.

## Authors’ information

Claudia Leone, International Public Health and Biostatistics Unit, Center for Malaria and other Tropical Diseases and WHO Collaborating Center for Health Workforce Policy and Planning, Instituto de Higiene e Medicina Tropical, Lisbon.

Cláudia Conceição,School of Health Sciences, University of Minho.

Gilles Dussault , International Public Health and Biostatistics Unit, Center for Malaria and other Tropical Diseases and WHO Collaborating Center for Health Workforce Policy and Planning, Instituto de Higiene e Medicina Tropical, Lisbon.

## Supplementary Material

Additional file 1: Table S1 Interviews in Portugal. Click here for file
