# Supplementary material for: Trends of cross-border mobility of physicians and nurses between Portugal and Spain
Source: Hum Resour Health. 2013 Jul 31;11:36. doi: 10.1186/1478-4491-11-36 (PMC3735413; doi:10.1186/1478-4491-11-36)
Supplement: Additional file 1: Table S1 — Interviews in Portugal. [file 1478-4491-11-36-S1.pdf]

**Additional Table 1– Interviews in Portugal**

| Thematic areas | Local Health Units or Hospitals Centre (*Centro Hospitalar*) | | | |
|---|---|---|---|---|
| | **Nordeste Hospital Centre (only hospital units)** | **Cova da Beira Hospital Center (only hospital units)** | **Norte Alentejano Local Health Unit (Hospital units and groups of health centres)** | **Baixo Alentejo Local Health Unit (Hospital units and group of health centres)** |
| **Characterization of professionals** | 18 physicians<br>12 Bragança<br>6 Mirandela<br><br>13 specialists<br>3 general practitioners<br>2 residents (internship)<br><br>General practitioners live in Spain and work in Portugal and specialists live in Portugal. | 7 nurses<br><br>12 physicians<br><br>8 hospital specialists<br>4 residents (interns)<br><br>Those are integrated and live in Portugal. | 44 physicians<br><br>In hospitals<br>17 specialists<br>2 residents (interns)<br><br>In Health Centres<br>23 general practitioners<br>2 residents (interns)<br><br>None of the general practitioners lives in Portugal. | 29 physicians<br><br>20 physicians: specialists in internal medicine and general practitioners<br>9 residents (interns)<br><br>Most live in Portugal. |
| **Reasons for mobility** | 1 – Professional development: to do a residency;<br>2 – Availability of positions in the interior of Portugal and better salaries;<br>3 – Better technical conditions to perform the work (team work, availability of surgery rooms,…) | 1 – Difficulty in getting job in Spain (at present improved and there is less mobility from Spain to Portugal);<br>2 – Availability of positions in the interior of Portugal and better salaries:<br>3 – This hospital is an accredited one and stands for high quality care. | 1 – Availability of positions in the interior of Portugal and better salaries (at present this reason is decreasing as better opportunities in Spain increase);<br>2 - Professional development: residency | 1 – Availability of positions in the interior of Portugal and better salaries |

**Additional table 1 – Interviews in Portugal** (contd)

| Thematic areas | Local Health Units or Hospitals Centre Complex (*Centro Hospitalar*) | | | |
|---|---|---|---|---|
| | **Nordeste Hospital Center Complex (only hospital units)** | **Cova da Beira Hospital Center Complex (only hospital units)** | **Norte Alentejano Local Health Unit (Hospital units and groups of health centres)** | **Baixo Alentejo Local Health Unit (Hospital units and groups of health centres)** |
| **Consequences of mobility** | Very positive view:<br>Improved supply of specialist care;<br>Helps address the problem of limited access to medical care | Very positive view:<br>Improved supply of specialist care;<br>Helps address the problem of limited access to medical care | Very positive view:<br>Improved supply of specialist care;<br>Helps address the problem of limited access to medical care | Very positive view:<br>Improved supply of specialist care;<br>Helps address the problem of limited access to medical care<br><br>Problem: language difficulties in patient-physician contact |
| **Incentives** | Increase of income with extra work in emergency room.<br>No incentive policy due to financial restrictions. | No incentive policy.<br>Offer of salaries equals the salaries of Public servants even if different kind of contract.<br>This is an accredited and University hospital and is among the first five better classified at national level. | | Accommodation offer limited for financial restrictions.<br>Access to a residency is also pointed as an incentive. |
| **Other** | | | Problems with degrees recognition.<br>Some lack of fairness perceived by professionals with regard to different types of contracts (and salaries) that present legislation allows. | |

**Additional Table 2 – Interviews in Spain**

| Thematic areas | Hospitals | | | |
|---|---|---|---|---|
| | **Hospital of Santisima Trinidad, Salamanca** | **Sanatorio of Santa María, Pontevedra** | **Juan Ramón Jimenez Hospital, Huelva** | **Miguel Dominguez Hospital, Pontevedra** |
| **Characterization of professionals** | 2 nurses | 12 nurses in recent years, 2 at the moment of the interview. | 1 nurse occasionally | 23 nurses<br><br>The organization started hiring Portuguese nurses in 2007<br><br>Most of them stay in Spain 1 or 2 years. |
| **Reasons for mobility** | 1 – Availability of positions. | 1 – Difficulty in getting job in Portugal<br>2 – Availability of positions. | 1 – Difficulty in getting job in Portugal<br><br>Mobility trends are stronger from Spain to Portugal. | 1 – Availability of positions.<br>2 – Difficulty in getting job in Portugal. |
| **Consequences of mobility** | Positive view:<br>It solves the problem of scarcity of medical care;<br>Portuguese professionals considered as having good attitudes and qualifications. | Positive view:<br>No negative consequences;<br>Portuguese professionals considered as having good attitudes and qualifications. | Positive view:<br>It solves the problem of scarcity of medical care;<br>No differences between Spanish and Portuguese professionals in terms of qualifications. | Positive view:<br>Portuguese professionals considered as having good attitudes and qualifications;<br>It solves the problem of scarcity of medical care |
